# Multifaceted Treatment Using Advanced Modalities for Refractory Achilles Tendinopathy: A Case Report

**DOI:** 10.7759/cureus.55030

**Published:** 2024-02-27

**Authors:** Toru Omodani

**Affiliations:** 1 Orthopaedics, Tokyo Advanced Orthopaedics, Tokyo, JPN

**Keywords:** ultrasound, prolotherapy, extracorporeal shock wave therapy, transarterial embolization, achilles tendinopathy, multidisciplinary treatment

## Abstract

Achilles tendinopathy is a prevalent issue among athletes, often resistant to traditional treatments, and can persist chronically. This report presents a 23-year-old female track athlete suffering from refractory Achilles tendinopathy for four years. Despite initial treatments including rehabilitation, the use of insoles, steroid injections, and extracorporeal shock wave therapy, her symptoms persisted. Implementing a combination of innovative treatments - transarterial embolization, extracorporeal shock wave therapy, and prolotherapy - resulted in a significant improvement in symptoms. The case underscores the potential efficacy of a multifaceted approach, suggesting that a combination of treatments may be essential for addressing the complex pathology of chronic Achilles tendinopathy.

## Introduction

Achilles tendinopathy affects two to three people out of 1,000 annually, and up to 50% of high-intensity runners develop this condition [1,2]. Generally, symptoms are believed to improve within three to 12 months, but it is not uncommon for chronic symptoms to persist for over 10 years [3,4]. Conservative treatments, such as rest, medication, physical therapy, injections, and extracorporeal shock wave therapy (ESWT), can be considered [5,6]. However, there is still no treatment method for refractory Achilles tendinopathy that is strongly recommended with clear evidence [7], making its management challenging.

In this report, we present a case in which combining advanced treatment options for refractory Achilles tendinopathy resulted in favorable clinical outcomes.

## Case presentation

A 23-year-old female track and field athlete who participates in heptathlon developed chronic pain in the left Achilles tendon area during competition without any traumatic episode. She was diagnosed with Achilles tendinopathy at a local hospital. Despite rest and rehabilitation for three months, there was no improvement. She also received treatments at other medical institutions, including the use of insoles, steroid injections around and inside her Achilles tendon, and extracorporeal shock wave therapy, but the improvement was minimal. Four years after the onset, she was referred to our clinic.

**Figure 1:**
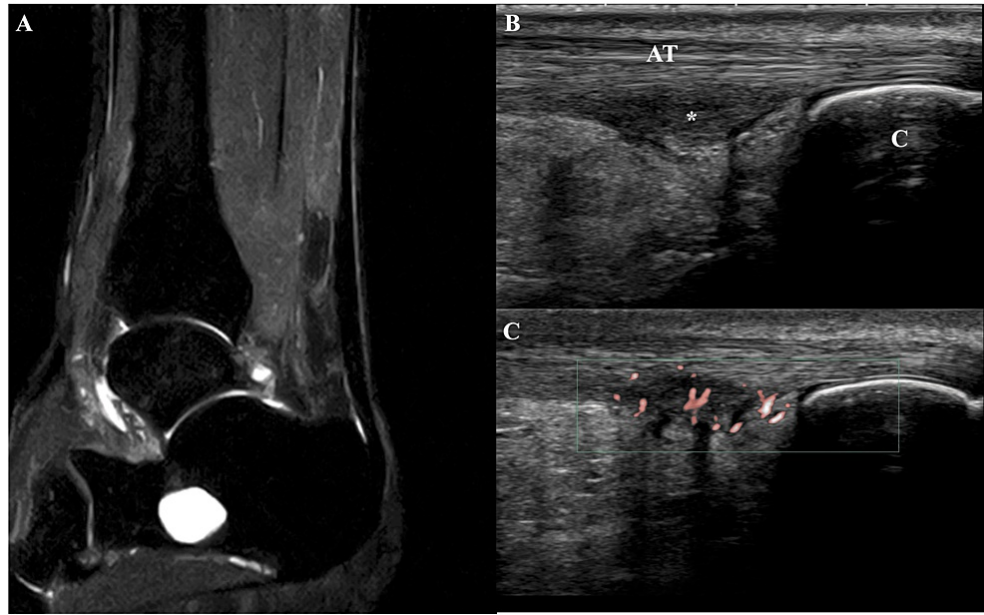
Imaging findings A: On the magnetic resonance imaging T2-weighted fat-suppressed images, thickening of the Achilles tendon substance was observed. B: Ultrasonography revealed a hypoechoic change in the distal and deep part of the Achilles tendon. C: Enhancement of the Doppler signal, suggesting increased blood flow. AT: Achilles tendon, C: calcaneus, asterisk: hypoechoic change

Mild swelling was observed in the substance of the Achilles tendon. There was no sensory deficit in the Achilles tendon area, and no increase or decrease in Achilles tendon reflex was noted. The range of motion of the ankle joint was 20 degrees in dorsiflexion and 45 degrees in plantarflexion, with no difference compared to the healthy side. The patient was able to perform a single-leg heel raise on the affected side; however, the movement was slower than on the healthy side due to pain in the Achilles tendon. Tenderness was observed from the midportion of the Achilles tendon to its insertion. On the MRI T2-weighted fat-suppressed images, a thickening of the Achilles tendon substance was observed (Figure 1A). Ultrasonography revealed a hypoechoic change in the distal and deep part of the Achilles tendon (Figure 1B). In addition, there was an enhancement of the Doppler signal, suggesting an increased blood flow (Figure 1C). The Japanese Society for Surgery of the Foot (JSSF) ankle/hindfoot score was 49 out of 100, and the sports section of the Self-Administered Foot Evaluation Questionnaire (SAFE-Q) score was 33.2 out of 100 [8,9]. It was decided to proceed with treatment using advanced treatment options that had not been applied to this patient before.

**Figure 2:**
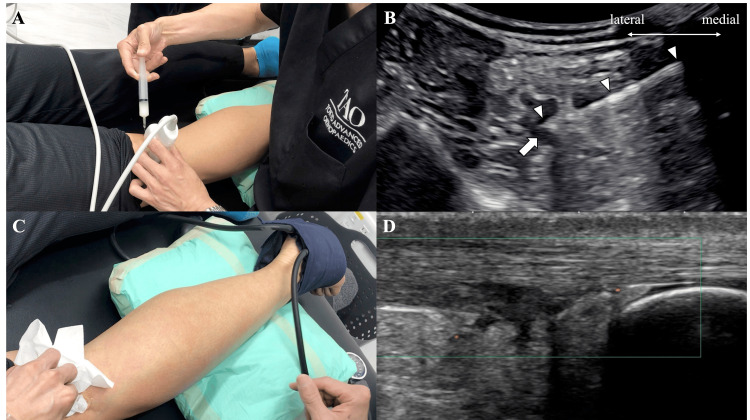
Transarterial embolization A, B: A 60 mm 22G needle was inserted from the medial side into the popliteal artery, and the embolizing agent was injected. C: After the injection, the skin of the lower leg turned pale, and reproducible pain in the Achilles tendon area was confirmed.

First, transarterial embolization was performed. The patient was placed in a prone position on the bed. A tourniquet was wrapped around the upper border of the Achilles tendon insertion and inflated to 300 mmHg. An ultrasound probe was placed in the short-axis direction on the posterior knee, and the popliteal artery was identified. A 60 mm 22G needle was inserted from the medial side into the popliteal artery, and the embolizing agent was injected (Figure 2A, 2B). As the embolizing material, 8 ml of a solution consisting of 0.5 g of imipenem dissolved in 10 ml of 10% dextran 40 was used. After the injection, the skin of the lower leg turned pale, and reproducible pain in the Achilles tendon area was confirmed (Figure 2C). About two minutes after the injection, the paleness and pain in the Achilles tendon peaked out, and the tourniquet was removed. When the Achilles tendon area was observed with ultrasound imaging immediately after the treatment, the Doppler signal observed before the treatment had almost disappeared (Figure 2D). No side effects, such as bleeding from the artery, nerve damage, or allergic reactions to the medication, occurred. The pain significantly improved after treatment, with the numerical rating scale (NRS) of pain dropping from 10 to 3. The intensity of running was restored up to 70%. However, while the pain in the midportion of the Achilles tendon almost disappeared, the pain at the tendon insertion became evident.

**Figure 3:**
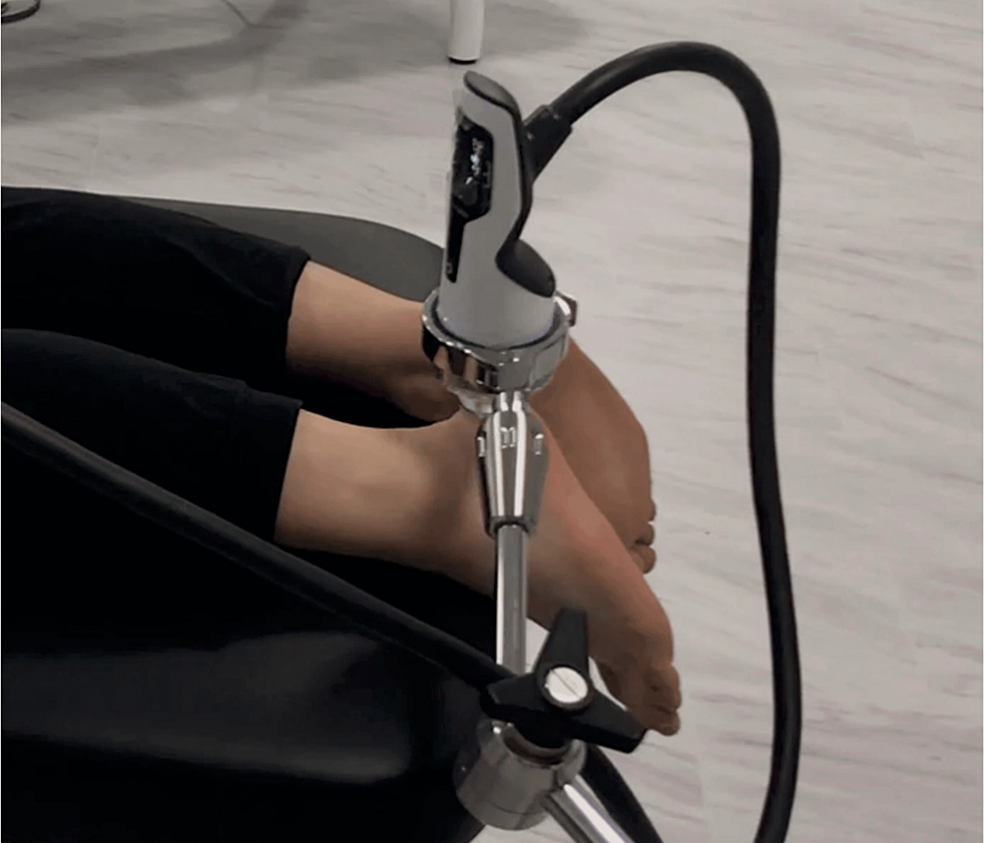
Extracorporeal shock wave therapy (ESWT) Focused ESWT was administered to the medial side of the Achilles tendon insertion.

A distinct localized tenderness was observed on the medial side of the Achilles tendon insertion. Focused extracorporeal shock wave therapy (ESWT) was administered to this area (Figure 3). Using the DUOLITH SD1 (STORZ MEDICAL AG: Switzerland), 2,500 shots were delivered at an energy of 0.25 mJ/mm^2^. After conducting two sessions of treatment at two-week intervals, the pain at the Achilles tendon insertion improved. The NRS decreased to 1, and her athletic performance in the heptathlon recovered to 90% of the level before the Achilles tendon was injured. However, slight pain remained in the substance of the Achilles tendon, which was also observed after the arterial embolization treatment.

**Figure 4:**
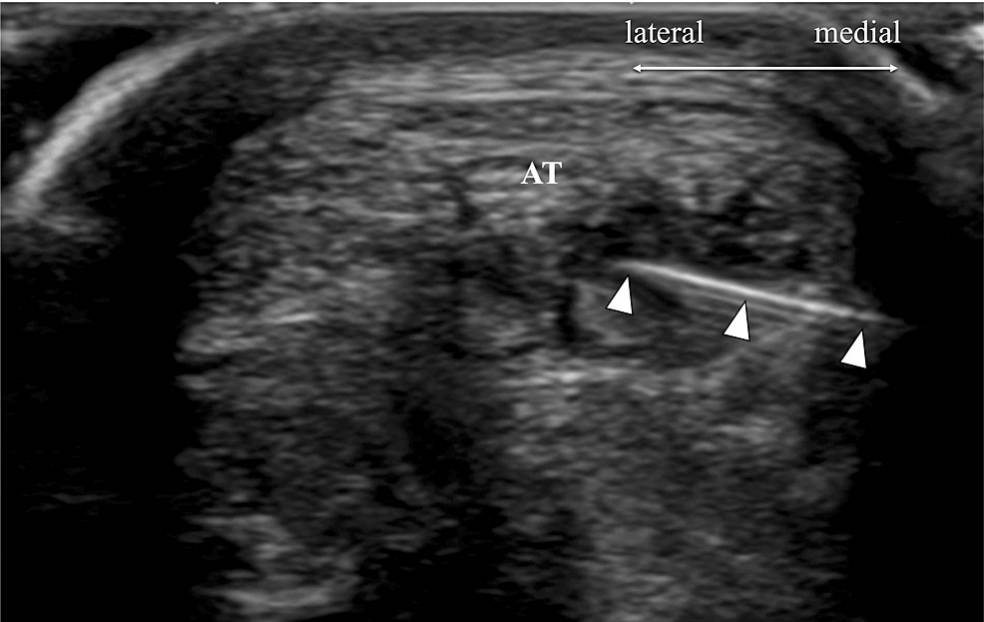
Prolotherapy 10% glucose solution was injected into the tendon where the hypoechoic area was depicted. AT: Achilles tendon, arrowheads: needle

Performing sonopalpation to examine tenderness while observing the ultrasound image, the hypoechoic area of the Achilles tendon was believed to be the cause of the remaining pain. Prolotherapy was administered to this area. A 10% glucose solution (1.5 ml), prepared by mixing with saline and lidocaine, was injected into the tendon where the hypoechoic area was depicted (Figure 4). After the injection, the slight pain that remained disappeared.

Three months after the start of treatment, the NRS for pain decreased to 0. The JSSF ankle/hindfoot score improved to 100 points, and the sports section of the SAFE-Q score improved to 99.4 points. Nine months after the start of treatment, there was no recurrence of symptoms, and the athlete achieved her long-held desire to compete in the national championships.

## Discussion

In this case, the patient had refractory Achilles tendinopathy that persisted for four years. However, by combining transarterial embolization, ESWT, and prolotherapy, we were able to achieve favorable clinical outcomes. The novelty of this case lies in the effective multifaceted approach that combined multiple treatment options, offering both unique insights and valuable perspectives.

Transarterial embolization is a new treatment method for musculoskeletal pain that has been difficult to improve with traditional treatments. Okuno was the first in the world to report that in lesions where chronic pain persists, abnormally fine vessels are newly formed and proliferate and that embolizing these vessels can alleviate pain [10]. The newly formed abnormal vessels are believed to proliferate and accompany unmyelinated nerve fibers, which contribute to pain [11]. It is thought that the mechanism by which transarterial embolization alleviates pain is by inducing necrosis of both the vessels and the associated nerve fibers [10]. The efficacy of transarterial embolization for chronic Achilles tendinitis has been reported [12,13]. Okuno reported a technique for transarterial embolization without the use of a catheter, and the method used in this case was inspired by that approach [14,15]. In this case, the reduction in Doppler signals suggesting blood flow and the improvement in pain achieved through transarterial embolization indicated its usefulness as a treatment approach.

ESWT is reported to be effective in alleviating pain by selectively destroying unmyelinated nerve fibers and inhibiting neurotransmitter transmission [16,17]. ESWT is believed to be most effective at interfaces where tissue acoustic impedance changes dramatically, making it well-suited for targeting areas where a tendon attaches to a bone. There have been reports indicating the effectiveness of focused ESWT for Achilles insertional tendinopathy [18,19]. In this case as well, focused ESWT proved effective for the pain at the calcaneal attachment of the Achilles tendon.

Prolotherapy is a general term for a treatment method that involves injecting an irritant to induce inflammation, which then promotes tissue repair and the activation of growth factors [20]. It is believed that the mechanism of sclerosing and shrinking abnormal blood vessels and accompanying unmyelinated nerve fibers using hyperosmolar solutions, as well as the anti-nociceptive effects of D-glucose, are effective in alleviating pain caused by tendinopathy [21,22]. The efficacy of prolotherapy for chronic Achilles tendinitis has been previously reported [23]. In this case, focused ESWT also effectively alleviated pain at the calcaneal attachment of the Achilles tendon. Steroid injections had already been administered to her Achilles tendon by a previous physician, and there was no conclusive evidence that any direct injection into the tendon would be effective. This was the reason for choosing transarterial embolization before prolotherapy.

By combining three treatments, we were able to alleviate the pain of refractory Achilles tendinitis. This suggests that the Achilles tendinopathy in this case may have involved a coexistence of multiple pathologies for which different treatments were effective. In addition, the fact that the degree of improvement in pain with each treatment was different suggests that there was a gradient in the proportion of these pathologies accounted for (Figure 5). The novelty of this report lies in the fact that a combination of different treatment options was performed and effective for multiple mixed pathologies. It appears that refractory Achilles tendon disorder may require a multifaceted approach that combines multiple treatment options. Such a combination therapy may be the key to future treatment of refractory Achilles tendinopathy.

## Conclusions

This report demonstrates the successful treatment of a refractory Achilles tendinopathy through a novel approach combining transarterial embolization, ESWT, and prolotherapy. The findings suggest that a tailored, multi-modal treatment approach may be essential for overcoming refractory Achilles tendinopathy. As such, this study offers a promising direction for the future management of similar cases.
